# Magnetic and Electrical Properties of CuCr_2_Se_4_ Nanoparticles

**DOI:** 10.3390/ma16237495

**Published:** 2023-12-04

**Authors:** Ewa Malicka, Tadeusz Groń, Adrian Gudwański, Bogdan Sawicki, Monika Oboz, Małgorzata Karolus, Zenon Kukuła

**Affiliations:** 1 Institute of Chemistry, University of Silesia in Katowice, 40-007 Katowice, Poland; ewa.malicka@us.edu.pl (E.M.); adrian.gudwanski@us.edu.pl (A.G.); 2 Institute of Physics, University of Silesia in Katowice, 40-007 Katowice, Poland; tadeusz.gron@us.edu.pl (T.G.); bogdan.sawicki@us.edu.pl (B.S.); monika.oboz@us.edu.pl (M.O.); zenon.kukula@us.edu.pl (Z.K.); 3 Institute of Materials Engineering, University of Silesia in Katowice, 40-007 Katowice, Poland

**Keywords:** high-energy ball milling, nanoparticles, magnetic measurements, electrical properties

## Abstract

CuCr_2_Se_4_ nanoparticles were obtained by the high-energy ball milling of CuCr_2_Se_4_ single crystals, which had a size of approximately 32 nm after 5 h of milling. Structural, magnetic, and electrical studies have shown that a reduction in CuCr_2_Se_4_ single crystals to the nanosize leads to (1) a weakening of ferromagnetic interactions, both long and short range, (2) a lack of saturation of magnetization at 5 K and 70 kOe, (3) a change in the nature of electrical conductivity from metallic to semiconductor, and (4) a reduction in the thermoelectric power factor S^2^σ by an order of magnitude of 400 K. The above results were considered in terms of the parameters of the band model, derived from the high-temperature expansion of magnetic susceptibility and from the diffusive component of thermoelectric power. Theoretical calculations showed a significant weakening of both the superexchange and double exchange mechanisms, a reduction in the [Cr^3+^,Cr^4+^] band width from 0.76 to 0.19 eV, and comparable values of the Fermi energy and the activation energy (0.46 eV) in the intrinsic region of electrical conductivity. The main advantage of high-energy ball milling is the ability to modify the physicochemical properties of already existing compounds for desired applications.

## 1. Introduction

Spinels with the general formula CuCr_2_X_4_ (where X = S, Se, and Te) are ferromagnetic [1,2] and metallic conductors at room temperature [3]. CuCr_2_X_4_ spinels doped with 3*d* metals show a transition to the ferrimagnetic or antiferromagnetic order and the semiconductor state. Many of them exhibit low lattice thermal conductivity of about 20 mW/cmK, independent of temperature, the conductivity of small polaron type in ferromagnetic semiconductors [4], and a crossover from positive to negative magnetoresistance [5]. The relationship between a material’s magnetic and electrical response is important in a variety of technological applications, including magnetic sensors, such as reading heads on computer hard drives and thermoelectric devices [4,5]. CuCr_2_Se_4_ obtained by solid-phase synthesis has a normal cubic type of structure with the symmetry of the $Fd\bar{3}m$ space group and the zero magnetic moment of the Cu ion in the tetrahedral site, whereas the three Bohr magnetons correspond with Cr^3+^ ions in the octahedral site [6]. Pure CuCr_2_Se_4_, both in monocrystalline and polycrystalline form shows strong ferromagnetic (FM) interactions in the long-range below the Curie temperature T_C_ = 460 K and short range, as evidenced by the high positive Curie–Weiss temperature θ = 465 K [1,2], as well as the electrical resistivity of 3.8 × 10^−6^ Ωm and the positive Seebeck coefficient of 20.5 µV/K at room temperature [3].

Interesting properties of CuCr_2_X_4_ spinel mono- and polycrystals became an inspiration for their research in the nanoscale [7,8,9,10,11]. CuCr_2_Se_4_ nanopinels were obtained using microwave-assisted polyol synthesis [7,8] or thermal decomposition and a reaction of mixed metal–oleate complexes with selenium in a high-boiling organic solvent [9,10,11]. It was established that (1) the Curie temperature of the nanocrystals exceeded the value obtained for a common bulk polycrystalline spinel sample [7,8], (2) the crystallites were ferromagnetic at room temperature and exhibited a low-temperature magnetic moment of about 2.3 µ_B_ per Cr, which is close to reported value for the bulk [9], and (3) increasing the size of the crystallites from 15 to 25 nm led to an increase in the Curie temperature from 395 to 413 K, which is still below the corresponding bulk value of 430 K. Also, saturation magnetization, Curie–Weiss temperature, and the effective magnetic moment turned out to be lower than the bulk [10]. (4) The anisotropic-shaped CuCr_2_Se_4_ nanocrystals were superparamagnetic near room temperature but exhibited FM behavior at lower temperatures, with magnetization values of 31 and 43 emu/g at 300 and 5 K, respectively [11]. The above studies show that the magnetic results of nanocrystals depend on the method and conditions of their preparation. The literature data on the magnetic parameters of the CuCr_2_Se_4_ bulk spinels also differ significantly. A common feature of bulk and nanosized CuCr_2_Se_4_ spinels is their ferromagnetism.

Our previous studies of the ZnCr_2_S_4_ [12] and ZnCr_2_Se_4_ [13] nanopinels synthesized by the high-energy ball milling (HEBM) method from elements and single crystals, respectively, showed a change from antiferromagnetic (AFM) to an ideal paramagnetic state for sulfur spinels and AFM in a spin glass state for selenium. In both cases, the nature of the electrical conductivity did not change.

Next, the related CuCr_2_S_4_ compound, synthesized by the HEBM method from the constituent elements Cu, Cr, and S [14] and CuS and Cr_2_S_3_ sulfides [15], showed a change in both the ordering of the magnetic moments from the ferromagnetic (FM) state to the antiferromagnetic (AFM) one, and the nature of the electrical conductivity from the metallic state for spinel microcrystallites to the semiconductor for spinel nanoparticles changed. The Curie (T_C_), Néel (T_N_), and Curie–Weiss (θ) temperatures, effective magnetic moment (µ_eff_), electrical resistivity (ρ), and the Seebeck coefficient (S) of CuCr_2_S_4_ spionels are collected in Table 1 for comparison.

The novelties of this work are electrical and magnetic studies of CuCr_2_Se_4_ nanoparticles obtained from single crystals of this compound by the HEBM method to nanosize. An additional reason for the research is the calculation of the band model parameters due to the fact that according to Philipsborn [16], covalence increases with an increase in the radius of anions in the sequence O, S, Se, Te and has a strong impact on the physical properties of materials.

## 2. Materials and Methods

### 2.1. Synthesis of Nanocrystals

Nanospinel CuCr_2_Se_4_ was obtained in the process of the high-energy ball milling of the single crystals of this compound. As in the case of ZnCr_2_Se_4_, CuCr_2_Se_4_ single crystals were obtained by gaseous chemical transport, using anhydrous chromium(III) chloride, CrCl_3_, as a transporting substance, according to the reaction equation:

4 CuSe + 2 CrCl_3_ ⇌ CuCr_2_Se_4_ + 3 CuCl_2_. (1)

**Figure 1:**
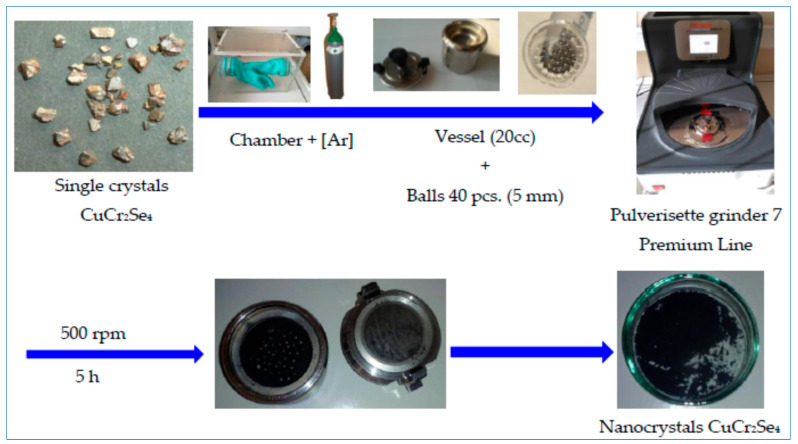
Block diagram of the synthesis of nanocrystalline CuCr_2_Se_4_.

The substrate in the synthesis process was copper(II) selenide*,* CuSe, obtained by a ceramic method by double sintering the copper and selenium powders at a temperature of 1073 K in quartz ampoules under vacuum conditions. Stoichiometric weights of substrates CuSe and CrCl_3_ were introduced into an ampoule made of quartz glass and were 20 cm long and 2 cm in diameter. The ampoule was sealed under vacuum using a turbomolecular pump at a pressure of 10*^−^*^5^ mbar. The gaseous chemical transport process was carried out for 16 days in a PRS-55HM horizontal tube-zone furnace. After the process was completed, the furnace was cooled to room temperature. After opening the ampoule in a hood, the wall surfaces of the obtained single crystals were cleaned by washing them several times with distilled water and cleaning them in acetone using an ultrasonic cleaner (INTERSONIC IS-1). The CuCr_2_Se_4_ single crystals obtained in the crystallization zone had an octahedral shape, and the edge length was about 0.5*–*4 mm (Figure 1).

The experimentally determined conditions for the synthesis of CuCr_2_Se_4_ single crystals, such as the number of substrates, the temperature of the crystallization and dissolution zone, the temperature gradient used (∆T), and the synthesis time are presented in Table 2.

In the second stage, the CuCr_2_Se_4_ single crystals were subjected to the process of high-energy milling. The ratio of the mass of single crystals to the mass of spheres was 1:10. The block diagram of the synthesis of nanocrystalline CuCr_2_Se_4_ is shown in Figure 1, and experimentally determined parameters of the milling process are presented in Table 3. The grinding time was selected based on our previous work with ZnCr_2_Se_4_ nanospinels, which was also obtained by grinding single crystals. In order to prevent the “decomposition” of the CuCr_2_Se_4_ compound, we experimentally ground it to a point where there were the fewest foreign phases (impurities). The phase analysis of the obtained CuCr_2_Se_4_ nanospinel was performed after 3 and 5 h of milling.

### 2.2. Methods

The static (dc) magnetic susceptibility was measured in the temperature range of 5–300 K and the field *H*_dc_ = 1 kOe and recorded both in zero-field-cooled (ZFC) and field-cooled (FC) mode. Magnetization isotherms were measured at 5, 20, 40, 60, and 300 K, while hysteresis loops were measured at 5, 10, 50, and 300 K using a Quantum Design MPMS-XL-7AC SQUID magnetometer (Quantum Design, San Diego, CA, USA) in applied external fields up to 70 kOe. The effective magnetic moment, µ_eff_, was calculated using the equation presented in Refs. [17,18]. Dynamic (ac) magnetic susceptibility was measured in the temperature range of 5 ÷ 300 K and at an internal oscillating magnetic field of *H*_ac_ = 3.9 Oe and internal frequency of *f* = 1 kHz using the QD-PPMS measurement system (Quantum Design Physical Properties Measurement System, Quantum Design, San Diego, CA, USA).

Electrical conductivity σ(T) of the samples under study was measured by the DC method using a KEITHLEY 6517B Electrometer/High Resistance Meter (Keithley Instruments, LLC, Solon, OH, USA) within the temperature range of 77–400 K. The thermoelectric power S(T), i.e., the Seebeck coefficient, was measured within the temperature range of 100–400 K with the help of a Seebeck Effect Measurement System (MMR Technologies, Inc., San Jose, CA, USA).

## 3. Results

### 3.1. Structural Characteristics

**Figure 2:**
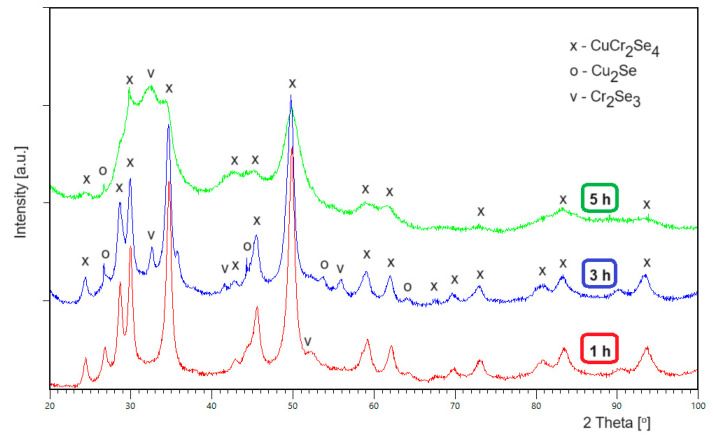
Diffractograms of a single-crystal CuCr_2_Se_4_ spinel after 1, 3, and 5 h of milling.

Figure 2 shows X-ray diffraction patterns obtained after 1, 3, and 5 h of milling single crystals of the CuCr_2_Se_4_ spinel. Phase analysis confirmed the presence of the CuCr_2_Se_4_ spinel phase (cubic system, space group $Fd\bar{3}m,$ ICDD PDF4+, card 04-004-0213) at every stage of the milling process. At the same time, in the diffraction pattern after 1 h of milling, a regular phase derived from Cu_2_Se was identified (space group $Fm\bar{3}m,$ ICDD PDF4+, card 04-003-4435), as well as trace amounts of a rhombohedral phase derived from Cr_2_Se_3_ (space group $R\bar{3},$ ICDD PDF4+, card 01-089-2068) after 3 h of the milling process. The mass fraction of the additional phases is small and does not exceed 3% by weight in the final material. The determined structural parameters of the CuCr_2_Se_4_ spinel subjected to the high-energy milling process are summarized in Table 4. With an increase in the milling time, we observe a regular increase in the lattice parameter to the value of *a* = 1.04139(8) nm after 5 h of milling and a systematic decrease in the size of the crystallites. The presence of significantly broadened reflections in the diffraction patterns after 5 h of milling indicates the nanocrystalline structure of the ground material, and then the nanocrystallites reach the smallest size of 32 nm, while the lattice strain is the largest and amounts to 0.85% (Table 4).

### 3.2. Electrical Properties

**Figure 3:**
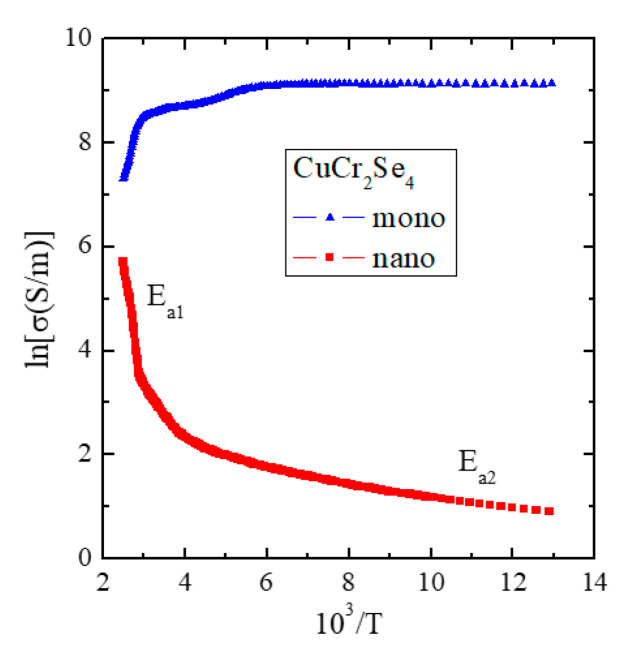
Electrical conductivity (lnσ) vs. reciprocal temperature (10^3^/T) of CuCr_2_Se_4_ mono- and nanospinels.

The results of the electrical conductivity measurements, σ(10^3^/T), of the CuCr_2_Se_4_ nanospinel clearly showed two areas: extrinsic in the temperature range of 77–160 K, in which the thermal activation E_a1_~0.01 eV is observed, and intrinsic in the temperature range of 350–400 K with a stronger thermal activation of E_a2_~0.46 eV (Table 5 and Figure 3). The value of σ at 400 K is ~3 × 10^2^ S/m and is five times lower compared to the CuCr_2_Se_4_ single crystal at the same temperature, from which the nanopinel was formed after milling, and at 77 K, this difference is four orders of magnitude (Figure 3).

The conductive CuCr_2_Se_4_ single crystal shows almost zero and negative activation energy in the extrinsic and intrinsic regions, respectively (Table 5). The reason for this may be dominant structural scattering and strong phonon scattering since the same type of potential is present. Phonon scattering, which is usually the dominant mechanism in simple metals, should increase linearly with temperature. This can be interpreted either with direct lattice scattering, i.e., potential scattering due to the random distribution of Cu and Cr atoms and/or vacancies and interstitial defects. In the CuCr_2_Se_4_ single crystal, this mechanism plays a significant role since a corresponding linear decrease in conductivity above room temperature is found (Figure 3). Similar behavior was found in the CuCr_2_Se_4_ single crystals doped with Ga [19] and Co [20]. In the case of the semiconductor CuCr_2_Se_4_ nanocrystal, the phonon mechanism is reduced as a linear increase in conductivity in the intrinsic region (Figure 3).

**Figure 4:**
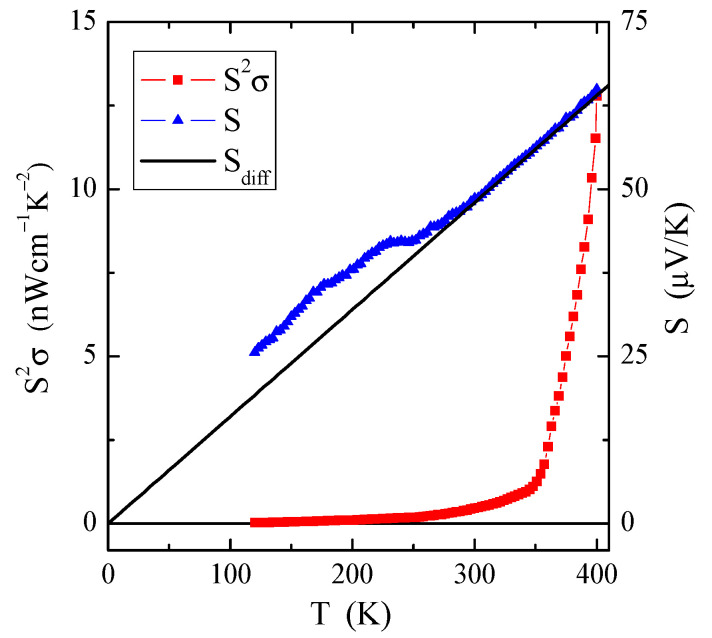
Thermoelectric power, S, and its diffusion component, S_diff_, as well as power factor, S^2^σ, vs. temperature T of the CuCr_2_Se_4_ nanospinel.

The temperature dependence of thermopower, S(T), measured for the nanocrystal under study, is depicted in Figure 4. Positive values of thermopower reveal hole-type electrical semiconductivity, which means that reducing the size of the crystalline conductor to a nanosize does not change the type of electrical conductivity, only its character. The S values at room temperature for the polycrystalline CuCr_2_Se_4_ spinel and nanospinel are 20.5 [3] and 48.6 µV/K, respectively.

The temperature dependence of thermoelectric power, S(T), presented in Figure 4, requires special consideration. In conventional metals, the thermopower consists of two different parts, i.e., a diffusion component (*S_diff_*), which according to the Mott formula [21] is proportional to temperature at high temperatures, and a phonon drag component (*S*_ph_), which is more complex. The *S_ph_* contribution results from a transfer of the phonon momentum to the electron gas. It drops both at low temperatures, such as *T*^3^ below θ_D_/10, when the phonons freeze out (where θ_D_ is the Debye temperature), and at high temperatures, such as *T*^−1^ above approximately θ_D_/2, when the phonon’s excess momentum is limited by anharmonic phonon–phonon scattering [22]. The literature values of the Debye temperature for CuCr_2_Se_4_ spinel crystals, ZnCr_2_Se_4_ nanospinels, and ZnCr_2_Se_4_:Re single crystals are 280 [23], 290 [12], and 295 K [24], respectively.

Figure 4 shows a rather well-defined linear slope of the thermoelectric power of the CuCr_2_Se_4_ nanospinel at high temperatures (marked by a solid line), which extrapolates to (0, 0). This diffusion part of the thermoelectric power (*S_diff_)* can be described by the Boltzmann electron transport equation [21] as follows:

(2) $$S_{diff}=\frac{\pi^{2}k^{2}}{eE_{F}}T=a\cdot T,$$

where *k* is the Boltzmann constant, *e* is the elementary charge, *E_F_* is the Fermi energy, and *a* is an empirical slope. In Equation (2), the Fermi energy, *E_F_*, can be written as follows:

(3) $$E_{F}=\frac{\pi^{2}k^{2}}{ea}.$$

Equation (3) allows us to evaluate the Fermi energy *E_F_* and the Fermi temperature *T*_F_ (defined as *E_F_/k*) using the experimental value of the slope of thermopower *a* for the polycrystalline CuCr_2_Se_4_ spinel [3] and the nanospinel under study. The values of *a*, *E_F_*, and *T_F_* are summarized in Table 5. In the case of the CuCr_2_Se_4_ polycrystal [3], the high-temperature part of the thermopower was extrapolated to (0, 0). Table 5 shows significant differences in *E_F_* and *T_F_* values between poly- and nanocrystalline spinels. Interestingly, in the case of the nanospinel, the values of *E_F_* and *E*_a1_ in the intrinsic region are identical. Compared to metals, e.g., for pure copper: *E_F_* = 7 eV and *T_F_* = 8.19 × 10^4^ K [25], non-metallic single crystal conductors, CuCr_2_Se_4_:Ga: *E_F_*~0.3 eV and *T_F_*~3 × 10^3^ K [19], single crystal semiconductors, ZnCr_2_Se_4_:Re: *E_F_*~0.047 eV and *T_F_*~550 K [24], and non-conductive single crystals, Pb_1−3x__x_Nd_2x_(MoO_4_)_1−3x_(WO_4_)_3x_: *E_F_*~0.042 eV and *T_F_*~490 K [26], the values of *E_F_* and *T_F_* of the studied nanospinels are typical for doped non-metallic spinel conductors.

Figure 4 also shows an interesting dependence of the power factor *S*^2^σ on temperature *T*. The value of *S*^2^σ increases significantly with increasing temperature, especially in the intrinsic region above 300 K, to reach 13 nWcm^−1^K^−2^ at 400 K. Compared to the studied CuCr_2_Se_4_ nanoparticles, *S*^2^σ at 400 K is by an order of magnitude higher in single-crystalline non-metallic spinel conductors CuCr_2_Se_4_:Ga [4,19], is of the same order as in single-crystalline spinel semiconductors ZnCr_2_Se_4_:Re [24], is five orders lower in weakly single crystals of lead molybdate–tungstates doped with neodymium [26], and is six orders lower in ceramic calcium molybdate–tungstates doped with Gd^3+^ and Co^2+^ [27] and calcium molybdate–tungstates doped with Nd^3+^ and Mn^2+^ [28]. The above studies show that thermoelectric efficiency is primarily determined by the type of chemical bond and electrical conductivity, and doping the matrixes with 3*d* and 4*f* metal ions can only slightly improve this efficiency. However, reducing the grain size to the nanoscale usually leads to a decrease in thermoelectric efficiency.

### 3.3. Magnetic Studies

**Figure 5:**
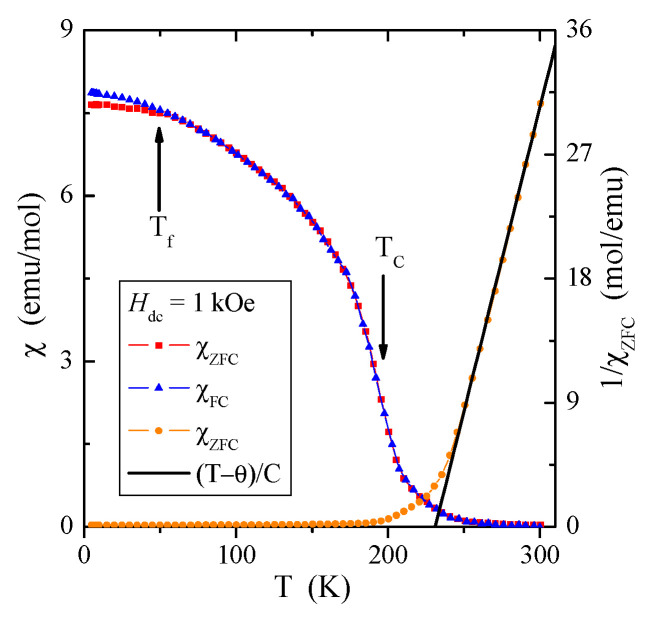
DC magnetic susceptibility $\chi_{ZFC},$ $\chi_{FC},$ $1/\chi_{ZFC}$, and (T − θ)/C (solid line) vs. temperature T of the CuCr_2_Se_4_ nanospinel.

**Figure 6:**
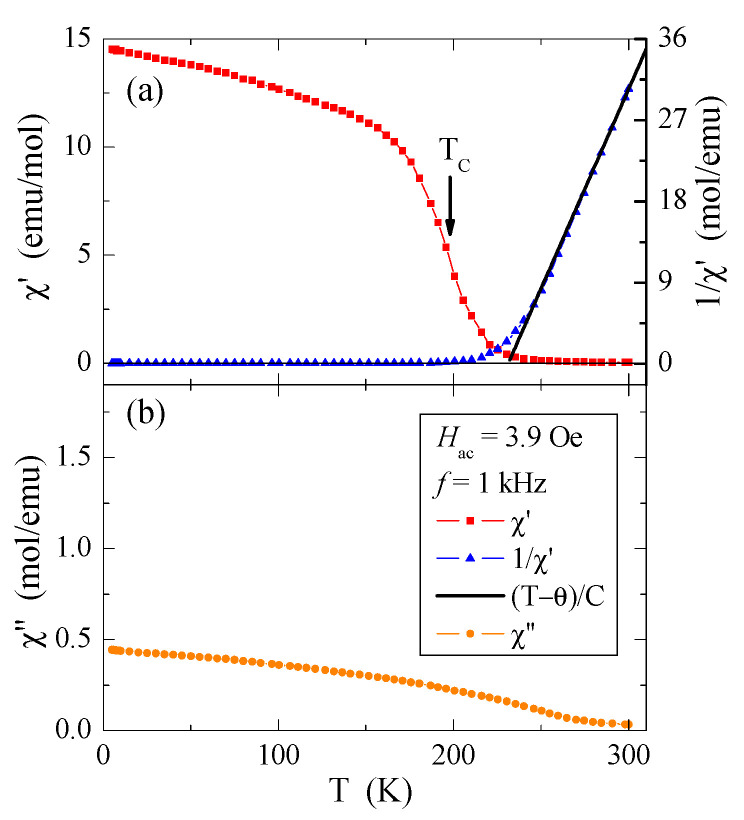
Real (**a**) and imaginary (**b**) part of AC magnetic susceptibility χ’, 1/χ’, (T − θ)/C (solid line), and χ’ vs. temperature T of the CuCr_2_Se_4_ nanospinel.

The results of DC and AC magnetic susceptibility measurements of the CuCr_2_Se_4_ nanspinel obtained by the HEBM method (*t* = 5 h, *d* = 32 nm) are presented in Table 6 and in Figure 5 and Figure 6a,b. The conducted studies have shown that the reduction in the size of crystallites leads both to the weakening of long-range ferromagnetic interactions, the Curie *T*_C_ temperature drops from 416 K [29] to 196 K, and the weakening of the short-range ferromagnetic interactions, as evidenced by the reduction in the paramagnetic Curie–Weiss temperature θ from 436 K [29] to 231 K (Table 6, Figure 5 and Figure 6a). In Figure 5, below the freezing point *T*_f_ = 50 K, there is a slight splitting of the DC susceptibility curves measured in the ZFC and FC mode, which may indicate the appearance of a geometric-type spin frustration characteristic in the spin glass state [30]. This is confirmed by the high energy loss visible in the imaginary magnetic susceptibility component, χ″, (Figure 6b), which is related to the spin reorientation and movement of the domain walls in the sample with a reduced grain size. Frustration may also result from the appearance of chromium ions with an oxidation state higher than 3+, as the effective magnetic moment, µ_eff_, of the investigated nanosipnel is smaller than the bulk (Table 6). The opposite behavior was observed for ZnCr_2_S_4_ spinel nanocrystals [12].

**Figure 7:**
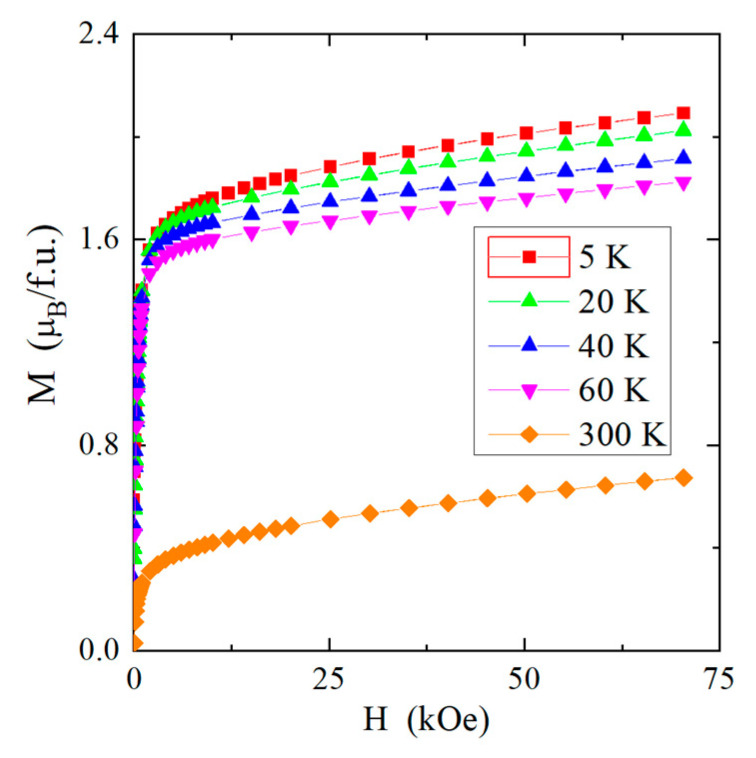
Magnetization M vs. magnetic field H at 5, 20, 40, 60, and 300 K of the CuCr_2_Se_4_ nanospinel.

The magnetic isotherms of the investigated nanopinel are shown in Figure 7. With the decrease in the size of the crystallites, the magnetization at 5 K in the magnetic field of 75 kOe strongly decreases from 4.76 μ_B_/f.u. for bulk to 2.09 μ_B_/f.u. for the nanospinel (Table 6), and magnetic isotherms move away from saturation at temperatures of 5, 20, 40, 60, and 300 K (Figure 7).

**Figure 8:**
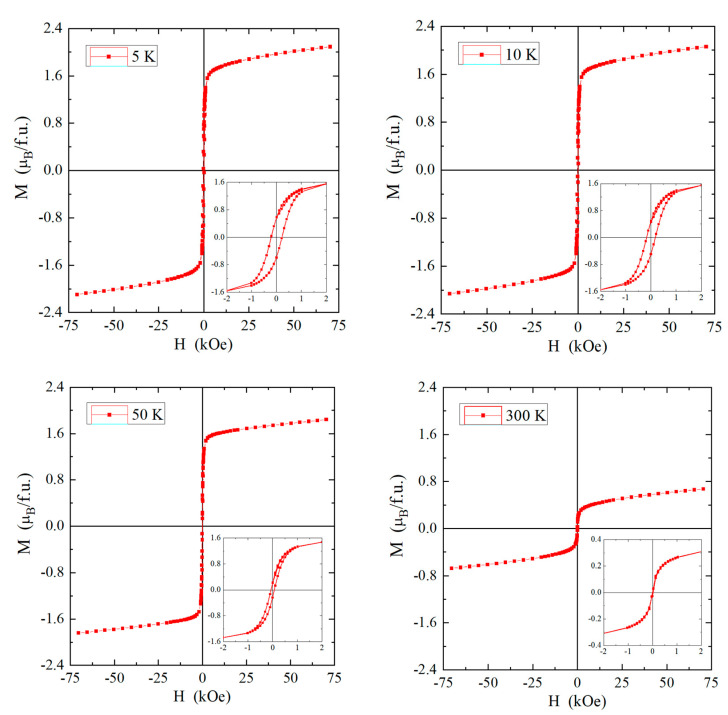
Hysteresis loops at 5, 10, 50, and 300 K of the CuCr_2_Se_4_ nanospinel. Insets: an enlarged magnetic hysteresis loop with visible coercive field and remanence.

At low temperatures, hysteresis loops are observed, which are characterized by relatively small values of the coercive field (Figure 8). For the lowest measuring temperature of 5 K, the coercive field is equal to *H*_c_ = 0.206 T (Table 6). An important feature characterizing magnetically disordered materials is the occurrence of magnetization hysteresis with a low value of remanence. In the studied nanospinel, the remanence value is low, approximately equal to *M*_r_ = 0.59 µB/f.u. At 300 K, we do not observe a hysteresis loop. The increasing energy of thermal vibrations decreases the value of the magnetization and the coercive field, and we observe a gradual shrinkage of the hysteresis loop until its disappearance after the transition from the ferromagnetic to the paramagnetic phase, i.e., when *T*_C_ > 196 K (Table 6). The confirmation of this transition in the *T*_C_ is the occurrence of a characteristic inflection point, visible both on the temperature curve of the DC magnetic susceptibility and the curve of the real part of the AC magnetic susceptibility (Figure 5 and Figure 6a).

## 4. Discussion

The explanation of the interesting properties of solid spinels of the AB_2_X_4_ type (where A = Cu, B = Cr, X = S, Se, and Te) was based on the Lotgering model [31], in which the copper ions are monovalent and form a filled Cu^1+^ band, which is located in tetrahedral sublattice (A), deep in the valence band. Then, in the octahedral sublattice (B), the corresponding number of chromium ions in the Cr^3+^ ($t_{2g}^{3}$) configuration changes into Cr^4+^ ($t_{2g}^{2}$), creating an unfilled mixed valence *t*_2g_ band of Cr^3+^-Cr^4+^ ions above the top of the valence band, which is responsible both for the high electrical conductivity and the strong ferromagnetic coupling of the chromium magnetic moments.

The type of magnetic interaction depends on the fact that the molecular field of the octahedron splits the energy levels of the magnetic ion by the value of Δ, the same as the molecular field of the tetrahedron with the reverse sequence [30]. The *e_g_* level is two-fold degenerated and the *t_2g_* level is three-fold degenerated. Δ = 10*Dq* is the distance between the splitting levels, where *Dq* is defined as the parameter of the molecular field and *D* is a constant of the potential of a cubic symmetry [30]. Electrons occupying these levels can stay in both low-spin and high-spin states. The magnetic contributions come from different magnetic interactions in spinels, mainly from the superexchange B^n+^-X-B^m+^ and B^m+^-X-X-B^n+^ interactions [31,32] between the localized spins expressed by the superexchange *J*_se_ (*J*_aa_ and *J*_ab_) integrals and the double exchange interactions [33] with the hopping integral *b*_de_ (*b*_aa_ and *b*_ab_) between the atomic *t*_2g_ states, without changing the spin orientation. In the first-order process (Zener [34]), there are two simultaneous motions (hence the name “double exchange”): the electron transfer from the B^3+^ cation to the X^2−^ anion and the electron transfer from the X^2−^ anion to the B^4+^ cation. Thus, the double exchange mechanism tends to align the site spins ferromagnetically [33]. The theoretical procedure for calculating the exchange integrals according to the Lotgering model [30] has already been described many times in works [29,35,36] and was used to calculate these integrals of the investigated nanopinel (Table 7 includes a bulk spinel for comparison) in order to interpret its electrical and magnetic properties.

The results presented in Table 7 show that reducing the grain size causes a two-fold weakening of the superexchange mechanism, a four-fold weakening of the double exchange mechanism, and almost a two-fold weakening of the effective interaction expressed by the J_aa_, b_aa_, and $J_{\mathrm{eff}}^{\mathrm{aa}}$ integrals for the first coordination zone, respectively. All these interactions are ferromagnetic. In the second coordination zone, both mechanisms are reduced and have anti- and ferromagnetic ordering, respectively. The consequence of this is a slight bifurcation of the ZFC and FC susceptibility below 50 K, which indicates a slight spin frustration (Figure 5). Thus, the spin frustration may be due to the competition of the FM and AFM interactions and not just the spin geometry, as the long-range (*T*_C_ = 196 K) and short-range (θ = 231 K) interactions are ferromagnetic. The hopping integral *B* and the associated band width *W*_d_ = 0.19 eV of the mixed valence band [Cr^3+^-Cr^4+^] strongly decrease with the size of the spinel nanoparticles. This primarily causes a change in the nature of electrical conductivity from metallic to semi-conductive (Figure 3) while maintaining the hole mechanism of *p*-type electrical conductivity (Figure 4). Differences in the values of magnetic parameters and exchange integrals visible in the example of CuCr_2_S_4_ [14] and CuCr_2_Se_4_ (Table 7) nanospinels are related to the strength of chemical bonds. According to Philipsborn’s suggestion [16], this is the result of an increase in the covalency of anions in the sequence O, S, Se, and Te. Narrowing the mixed valence band of chromium ions to 0.19 eV creates an energy gap between the bottom of this band and the top of the valence band. The Fermi level is at a distance of 0.46 eV from the top of the valence band and is equal to the activation energy in the intrinsic region of electrical conductivity of the nanospinel (Table 5).

## 5. Conclusions

It has been shown that the method of high-energy milling of single crystals is an effective method of obtaining single-phase nanospinels. Selenium nanopinels present a homogeneous phase without impurities. Reducing the size of single crystals to nanosize resulted in (1) a shift in the ferromagnetic long- and short-range interactions toward lower temperatures by about 200 K, (2) a change in the nature of *p*-type electrical conductivity from metallic to semiconducting, and (3) a decrease in the thermoelectric power factor to 13 nWcm^−1^K^−2^ at 400 K. The parameters of the band model determined from (1) measurements of the diffusive component of the thermoelectric power and calculations based on Mott’s theory showed that the Fermi energy perfectly correlates with the value of the activation energy in the intrinsic region of the electrical conductivity and (2) calculations of the magnetic exchange integrals derived from the high-temperature expansion of magnetic susceptibility showed a reduction in the width of the mixed valence band of chromium ions to the value of 0.19 eV, caused by the appearance of an energy gap between the bottom of this band and the top of the valence band. These studies have shown that the electrical and magnetic properties of solid materials can be modified to obtain desired applications by reducing the size of their grains. The strength and type of chemical bond have a significant impact on this modification.

## Figures and Tables

**Table 1:** Magnetic and electrical parameters of CuCr_2_S_4_ bulk [1,2] and nanospinels obtained from elements [14] and sulfides [15].

CuCr_2_S_4_	T_C_, T_N_ (K)	θ (K)	µ_eff_ (µ_B_/f.u.)	ρ (Ωm)	S (µV/K)	Ref.
bulk	420	390	4.4	4 × 10^−4^	+16.0	[1,2]
nano	30	198	3.53	1.8	+63	[13]
nano	40	258	1.934	2.13	−767	[14]

**Table 2:** Conditions for the synthesis of CuCr_2_Se_4_ single crystals.

Substrate Weigh (g)		Zone Temperature (K)		∆T (K)	Time (h)
CuSe	CrCl_3_	T_disso_.	T_cryst_.	90	384
4.5605	2.5335	1023	933		

**Table 3:** Parameters of the CuCr_2_Se_4_ high-energy milling process.

Mill Type	Pulverisette 7
Vessel capacity	20 cc
Milling balls	40 balls (diameter 5 mm, weight 0.51 g each)
Milling rotation	500 rpm
Milling time	15 min
Cooling	15 min
Number of cycles	20
Total milling time	5 h

**Table 4:** Structural parameters of the CuCr_2_Se_4_ nanopinel.

t (h)	a (nm)	d (nm)	η (%)
1	1.03719(6)	119	0.49
3	1.04090(4)	107	0.47
5	1.04139(8)	32	0.85

t is the milling time, a is the unit cell parameters, d is the size of crystallites, and η is the lattice strain.

**Table 5:** Electrical parameters of the CuCr_2_Se_4_ spinel in bulk and nanosize.

CuCr_2_Se_4_	E_a1_ (eV)	E_a2_ (eV)	a (µV/K^2^)	E_F_ (eV)	T_F_ (K)
bulk	<0	≈0	0.036 ^(^*^)^	2.04 ^(^*^)^	23622 ^(^*^)^
nano	0.46	0.01	0.16	0.46	5315

E_a1_ and E_a2_ are the activation energies at the intrinsic and extrinsic regions, respectively, a is the slope of the linear S_diff_(T) diffusion function of thermopower, E_F_ is the Fermi energy, T_F_ is the Fermi temperature. ^(^*^)^ These values were estimated from the S(T) data in Ref. [3].

**Table 6:** Magnetic parameters of the CuCr_2_Se_4_ bulk and nanospinels.

CuCr_2_Se_4_	C (emu·K/mol)	T_C_ (K)	T_f_ (K)	θ (K)	μ_eff_ (μ_B_/f.u.)	H_c (5K)_ (T)	M_r(5K)_ (μ_B_/f.u.)	M_s(5K)_ (μ_B_/f.u.)
bulk	2.70	416	-	436	4.65	-	-	4.76
nano	2.27	196	50	231	4.26	0.206	0.59	2.09

C is the Curie constant, T_C_, T_f_, and θ are the Curie, freezing, and Curie–Weiss temperatures, respectively, µ_eff_ is the effective magnetic moment, H_c_ is the coercive field, M_r_ is the remanence, and M_s_ is the saturation magnetization at 5 K. The values of magnetic parameters for bulk were taken from Ref. [29].

**Table 7:** Calculated magnetic parameters for superexchange and double exchange interactions of the CuCr_2_Se_4_ nanosipnel.

Spinel	x_3_ (Cr^3+^)	x_4_ (Cr^4+^)	X	θ_se_ (K)	θ_de_ (K)	B (K)	W_d_ (eV)	J_aa_ (K)	J_ab_ (K)	b_aa_ (K)	b_ab_ (K)	$J_{\mathrm{eff}}^{\mathrm{aa}}$(K)	$J_{\mathrm{eff}}^{\mathrm{ab}}$(K)
bulk	0.512	0.488	2.896	90	340	4391	0.76	63.9	−9.65	332.65	65.53	466.51	−83.5
nano	0.64	0.36	3.12	100	131	1093	0.19	31.1	−4.1	82.80	16.56	205.68	−41.7

x_3_ and x_4_ are the portions of the Cr^3+^ and Cr^4+^ ions, respectively, X is the mixture of spins due to the presence of chromium ions, θ_se_ is the superexchange contribution to θ, θ_de_ is the double exchange contribution to θ, B is the total hopping integral for the first and second coordination spheres, W_d_ is the mixed valence (Cr^3+^,Cr^4+^) bandwidth, J_aa_ and J_ab_, b_aa_ and b_ab_, and $J_{\mathrm{eff}}^{\mathrm{aa}}$ and $J_{\mathrm{eff}}^{\mathrm{ab}}$ are the superexchange, double exchange, and effective integrals for the first two coordination spheres, respectively. The values for bulk are taken from Ref. [29] for comparison.

## Data Availability

The data presented in this study are available on request from the corresponding author.
